# Identifying barriers to and facilitators of tuberculosis contact investigation in Kampala, Uganda: a behavioral approach

**DOI:** 10.1186/s13012-017-0561-4

**Published:** 2017-03-09

**Authors:** Irene Ayakaka, Sara Ackerman, Joseph M. Ggita, Phoebe Kajubi, David Dowdy, Jessica E. Haberer, Elizabeth Fair, Philip Hopewell, Margaret A. Handley, Adithya Cattamanchi, Achilles Katamba, J. Lucian Davis

**Affiliations:** 1 Uganda Tuberculosis Implementation Research Consortium, College of Health Sciences, Makerere University, Kampala, Uganda; 2 Department of Social and Behavioral Sciences, School of Nursing, University of California, San Francisco, CA USA; 3 Child Health and Development Centre, School of Medicine; College of Health Sciences, Makerere University, Kampala, Uganda; 4 Department of Epidemiology, Johns Hopkins Bloomberg School of Public Health, Baltimore, Maryland USA; 5 Center for Global Health, Massachusetts General Hospital, and Harvard University Medical School, Boston, MA USA; 6 Division of Pulmonary and Critical Care Medicine, and Curry International Tuberculosis Center, San Francisco General Hospital, University of California, San Francisco, CA USA; 7 Department of Biostatistics and Epidemiology, School of Medicine, University of California, San Francisco, CA USA; 8 Division of General Internal Medicine, San Francisco General Hospital, University of California, San Francisco, CA USA; 9 Clinical Epidemiology Unit, Department of Medicine, School of Medicine, College of Health Sciences, Makerere University, Kampala, Uganda; 10 Department of Epidemiology of Microbial Diseases, School of Public Health, and Pulmonary, Critical Care, and Sleep Medicine Section, School of Medicine, Yale University, New Haven, CT USA

**Keywords:** Tuberculosis, Household contact investigation, Lay health workers, COM-B model, Behavior Change Wheel framework, Implementation science

## Abstract

**Background:**

The World Health Organization recommends routine household tuberculosis contact investigation in high-burden countries but adoption has been limited. We sought to identify barriers to and facilitators of TB contact investigation during its introduction in Kampala, Uganda.

**Methods:**

We collected cross-sectional qualitative data through focus group discussions and interviews with stakeholders, addressing three core activities of contact investigation: arranging household screening visits through index TB patients, visiting households to screen contacts and refer them to clinics, and evaluating at-risk contacts coming to clinics. We analyzed the data using a validated theory of behavior change, the Capability, Opportunity, and Motivation determine Behavior (COM-B) model, and sought to identify targeted interventions using the related Behavior Change Wheel implementation framework.

**Results:**

We led seven focus-group discussions with 61 health-care workers, two with 21 lay health workers (LHWs), and one with four household contacts of newly diagnosed TB patients. We, in addition, performed 32 interviews with household contacts from 14 households of newly diagnosed TB patients. Commonly noted barriers included stigma, limited knowledge about TB among contacts, insufficient time and space in clinics for counselling, mistrust of health-center staff among index patients and contacts, and high travel costs for LHWs and contacts. The most important facilitators identified were the personalized and enabling services provided by LHWs. We identified education, persuasion, enablement, modeling of health-positive behaviors, incentivization, and restructuring of the service environment as relevant intervention functions with potential to alleviate barriers to and enhance facilitators of TB contact investigation.

**Conclusions:**

The use of a behavioral theory and a validated implementation framework provided a comprehensive approach for systematically identifying barriers to and facilitators of TB contact investigation. The behavioral determinants identified here may be useful in tailoring interventions to improve implementation of contact investigation in Kampala and other similar urban settings.

**Electronic supplementary material:**

The online version of this article (doi:10.1186/s13012-017-0561-4) contains supplementary material, which is available to authorized users.

## Background

In recent years, tuberculosis (TB) programs worldwide have seen modest declines in TB incidence and mortality [1], yet TB remains a major global cause of death and disability [2], especially in the 30 high-burden countries which account for more than 80% of TB patients worldwide [3]. Furthermore, recent projections show that the traditional, passive facility-based approach to TB case finding cannot begin to achieve the World Health Organization’s (WHO) goal of eliminating TB as a public health threat by 2035, defined as reducing annual TB incidence to below 10 per 100,000 [4]. The traditional, “passive” TB case-finding strategy depends on widespread awareness of TB symptoms and early clinic attendance by affected individuals. Unfortunately, these features are rarely observed in high TB-burden communities, with the result that infectious patients remain in the community longer, thereby worsening individual outcomes and perpetuating the cycle of airborne, person-to-person transmission of TB [5–7]. Thus, there is an urgent need to establish “active” TB case-finding programs in community settings [4].

The prototypical community-based active case-finding strategy is household contact investigation, in which health workers visit homes of patients with newly identified TB to screen co-habitants or close contacts for TB and refer at-risk individuals to clinics for evaluation and treatment [8]. In 2012, the WHO formally recommended that household contact investigation be routinely performed in high-burden countries [9], but few countries have systematically implemented this intervention [10]. Expert guidelines on how to implement household contact investigation in these settings have recently been published [11], but there is an ongoing need for research into barriers to and facilitators of contact investigation to better inform uptake.

Behavioral theory can inform the design of behavior-change interventions [12, 13], and there is growing evidence that this approach helps tailor interventions to context-specific barriers leading to improved results [14–19]. The capability, opportunity, and motivation determine Behavior (COM-B) model and its linked implementation framework, the Behavior Change Wheel (BCW), is one such theory. Developed from a systematic review of 19 existing theories of behavior change [14, 20], COM-B and BCW provide a comprehensive and coherent approach to implementation that has been widely applied in clinical and public health settings for behavioral analysis and intervention development [14–16, 21–23]. COM-B identifies three universal determinants of behavior—“capability” (sub-divided into physical and psychological capability), “opportunity,” (sub-divided into physical and social opportunity), and “motivation” (sub-divided into automatic and reflective motivation)—and links them to nine intervention functions of the BCW, each targeting one or more of these determinants [20].

COM-B and BCW stipulate that changing behavior requires changing these underlying determinants. Capability represents the aptitude to engage in a behavior and has physical (e.g., strength, skills, stamina) and psychological (e.g., knowledge, memory) domains. Opportunity represents environmental factors that affect the capability to perform a behavior and has physical (e.g., time, physical environment) and social (e.g., interpersonal influences, social cues, cultural norms) domains. Finally, motivation represents internal factors that allow one to employ capability and opportunity to perform a behavior; motivation has “automatic” (e.g., wants, needs, impulses) and “reflective” (e.g., beliefs, intentions) domains [14, 20, 21]. Applying the COM-B model helps formulate a “behavioral diagnosis” for a problem, and the BCW framework then allows systematic identification of named interventions targeting each domain of the behavioral diagnosis. For example, “education” addresses psychological capability barriers such as lack of knowledge or belief; “environmental restructuring” addresses physical opportunity barriers such as inefficient clinic processes; and “persuasion” addresses reflective motivation barriers such as reluctance to disclose one’s diagnosis to family members [13].

In previous research on TB evaluation and diagnosis in primary health centers in Uganda, we have explored barriers to delivery of patient-centered care [24–27], one of three pillars of the new WHO END TB Strategy [4]. These studies have collectively sought to test the general hypothesis that systematic application of behavioral theory during implementation can enhance delivery of evidence-based diagnostic strategies by adapting them to fit the needs of patients and providers in the local context. Here, we continue this line of investigation, reporting on a qualitative study in which we applied the COM-B model to identify and classify, in behavioral terms, the facilitators of and barriers to household TB contact investigation. We then used the BCW framework to identify potential interventions targeted to these behavioral determinants with the goal of improving the implementation of contact investigation.

## Methods

### Setting

Uganda is one of 30 WHO-designated, high TB-burden countries, with an estimated annual TB incidence of 161 per 100,000 and a TB case-detection rate of 72% in 2014 [1]. The Uganda National TB and Leprosy Programme (NTLP) is responsible for all TB control activities in Uganda. National TB guidelines provide a high-level recommendation that TB contact investigation should be routinely performed [28]; but no specific local guidelines about how to perform contact investigation are available. However, in January 2014, the NTLP and community partners introduced household TB contact investigation at all basic TB treatment units in Kampala, with household visits carried out by paid lay health workers (LHWs) already assigned to provide TB treatment-adherence support to TB patients in their homes.

To support the efforts of the Uganda NTLP to improve TB diagnostic evaluation and case detection, we established a community-based network for implementation research at seven public health facilities in Kampala; six out-patient facilities affiliated to the Kampala Capital City Authority (KCCA) and one local general hospital. These facilities provide free primary health care, as well as TB and HIV evaluation and treatment through specialized nursing units with on-site, quality-assured laboratories. They are staffed by medical doctors, clinical officers (clinicians who have completed a two-year diploma in medicine), nurses, laboratory technicians, and other trained health assistants; there are also positions, usually unpaid, for LHWs. The LHWs come from the communities served by the health facility to which they are attached. They are trained to educate patients and their families about TB and HIV, and to identify and refer symptomatic and high-risk household contacts to health centers. LHWs are supervised by both a LHW supervisor and a NTLP-designated TB focal person based at each health facility.

### Study design and population sample

We carried out a total of 10 focus groups and 32 interviews at the health centers within our network and at index patient homes between February and November 2014. We targeted three groups of stakeholders [11] who play key roles in household contact investigation: clinic-based health-center staff; clinic-affiliated LHWs; and adult household contacts of index patients. We held one focus group per clinic for the health-center staff, two focus groups for LHWs, and one for one household with four contacts present. In the other households, we interviewed contacts individually.

Between February and May, 2014, consent was obtained from all 61-available staff at the seven health centers to participate in facility-based focus groups (one per center). Between September and November, 2014, an additional 21 of 22 (95%) LHWs affiliated with these health centers consented to participate in one of two additional focus groups; one LHW was unavailable. From October to November 2014, 14 consecutive index TB patients with one or more household contacts were approached; all 14 consented to household visits. The visits identified 36 contacts (range 1–8 per household); all consented. Conversations lasted 60–90 min with health-center staff; 90 min with LHWs; and 30–60 min with contacts.

### Research team

After participating in 4 h of training led by an anthropologist with extensive experience in qualitative research (SA), local research staff conducted focus group discussions (FGD) and interviews in settings familiar to participants. The team included a social scientist and several experienced TB researchers who were familiar with WHO guidelines on TB contact investigation and had previously conducted TB research together [29–34]. A doctoral-trained social scientist (PK) led initial focus group discussions with health-center staff. Subsequently, a bachelors-trained social scientist (JG) led focus group discussions and interviews with LHWs and contacts. A team of one or more medical officers (IA, PH, IK) and a laboratory technician (EO) attended all sessions to take notes and ask TB-specific follow-up questions.

### Recruitment

All health-center staff, including those not directly involved in contact investigation and LHWs at all seven health centers were eligible for the study and were invited to attend focus groups. These sessions were held in the clinics during work hours reserved for administrative activities. For contacts, a consecutive sample of households of new adult index TB patients referred by LHWs was visited after verbal consent was obtained from the index patient. To obtain a wide range of perspectives, households were randomly assigned to have data collected before or after contact investigation was conducted. Households were recruited until data saturation was achieved; defined as the point at which each subsequent interview no longer yielded new themes [35]. The first round of visits to 10 households was conducted and these interviews identified an initial set of dominant themes. A second round of four household visits was then carried out. When the second round confirmed the earlier themes without introducing new insights, it was determined that we had attained saturation.

### Data collection and study instruments

To establish an empirical evidence base and to facilitate data collection on implementation of household contact investigation, separate interview guides for each of the three stakeholder groups were developed (Additional file 1). Each included six to eight open-ended questions exploring barriers and facilitators of contact investigation and associations between behavioral determinants and completion of three key TB contact investigation activities identified in conversations with the NTLP Program Manager and Zonal TB Supervisor: arranging household visits through index TB patients; visiting households to screen contacts for TB symptoms and TB risk factors and refer them to health centers; and performing clinical assessment and TB diagnostic testing of all at-risk contacts at health centers (Table 1). Interview and discussion guides were drafted in English, and piloted and refined with a convenience sample of non-participating health workers. A professional interpreter translated the guide for contacts into Luganda, the local language, and these were reviewed by the field research team (all of whom were bilingual) for accuracy; and were piloted and refined. The same guides were used for both the focus groups and interviews. Before each session, the facilitator introduced the research team and explained our objectives. The team took notes and summarized findings for participants at the end of each session. Following data collection, the project manager (IA) and a social scientist (JG) prepared summary reports of all notes. All focus group discussions and in-depth interviews were audio-recorded with participant permission and the research team had the recordings professionally transcribed.

**Table 1:** Principal stakeholders, activities, and individual behaviors involved in household TB contact investigation

Stakeholders	Activities		
	Arrange household visits	Visit households to screen contacts	Evaluate contacts in clinics
Clinic-staff	Identify index patients		Evaluate referred contacts
	Educate index patients		Perform diagnostic testing
	Obtain list of household contacts		Prescribe treatment
Lay health workers (LHWs)	Schedule a time for the visit	Educate household contacts	
	Obtain directions to the household	Interview household contacts	
		Refer eligible contacts to clinic	
Index patient	Consent or not to household visit		
	Provide directions to the household		
Household contacts		Consent to screening	Visit clinic after being referred
		Accept or not accept TB education	
		Answer TB screening questions	

### Analysis

Study investigators (AC, LD) debriefed the research team between sessions, and supervised them in categorizing emergent themes. We developed a coding tree with parent categories for the three main activities of contact investigation, and child categories based on barriers and facilitators elicited for each activity. Next, we further categorized barriers and facilitators by their associated behavioral determinants using the COM-B model [14, 20]. Finally, using the BCW framework, we identified functions that interventions should serve in order to alleviate barriers to and promote facilitators of the three core contact investigation activities. Two individuals (IA, JG) coded all transcripts using Dedoose, Version 6, (SocioCultural Research Consultants, Manhattan Beach, CA), an online application for collaborative qualitative data analysis. All coding differences were discussed, and if not resolved by discussion, were referred to an expert social scientist (SA) for arbitration.

### Human subjects’ protection

All participants provided informed consent. The School of Medicine Research and Ethics Committee at the Makerere College of Health Sciences, the Uganda National Council for Science and Technology, the Committee on Human Research at the University of California San Francisco, and the Human Investigation Committee at Yale University approved the study.

## Results

### Demographic characteristics of study participants

Participating health-center staff included 37 nurses from different categories (nursing officers, enrolled nurses, nursing assistants), five medical officers, seven clinical officers, five lab technicians, two counsellors, three pharmacy technicians, one data officer, and one multi-clinic LHW supervisor. Years of service in the current health center post ranged from one to 15 years (median 1 year; IQR 3 months—1 year). Total years of service for the LHWs ranged from one to twenty years (median 4 years; IQR 2–7). Median age of contacts was 33 (IQR 25–40) years. Women represented 40 (66%) of 61 health-center staff participants, 16 (76%) of 21 LHWs, and 19 (53%) of 36 household contacts. The following section highlights barriers to and facilitators of contact investigation.

### Arranging household visits through index TB patients: barriers

The first activity in TB contact investigation is arranging a household visit, which includes several key steps: identifying index patients, obtaining permission to visit households, arranging a time to visit, and traveling to and locating households. Themes that emerged as barriers to arranging home visits included (1) shortages of staff available to explain contact investigation to patients and to visit households; (2) lack of dedicated clinic space for TB care, particularly private places for counselling; (3) fear of contracting TB among staff; and (4) fear of stigma among index patients. The barriers identified throughout the three key contact investigation activities are organized within the COM-B framework in Table 2A.

**Table 2:** Barriers and facilitators of key household TB contact investigation activities in terms of their behavioral determinants

Behavioral determinant	Arranging home visits	Visiting households to screen contacts	Evaluating contacts in clinics
A. Barriers			
Capability^a^			
Psychological		Lack of TB knowledge among contacts	Lack of local contact investigation guidelines
		Language barrier for LHWs and contacts	
Physical			
Opportunity^b^			
Physical	Insufficient personnel at TB unit	Difficulty locating households	Lack of funds for travel for contacts
	Lack of dedicated clinic space for TB care	Difficulty finding contacts at home	
Social	Stigma felt by index patients	Avoidant behaviors of contacts	Stigma felt by contacts
Motivation^c^			
Automatic	Fear of getting TB among clinic staff	Fear of TB diagnosis among contacts	
Reflective			Distrust of clinic- staff among contacts
B. Facilitators			
Capability			
Psychological	Interpersonal skills of LHWs		
	Ability of LHWs to persuade index patients		
Physical			
Opportunity			
Physical	Task shifting to LHWs	Flexible scheduling of home evaluation	Streamlining contact evaluation at clinic
	Communication with patients via mobile phones	Fare for transport of LHWs to homes	Family physical support for contacts
Social	Trust between index patients and LHWs	Trust between contacts and LHWs	Family social support for contacts
		Privacy provided by home evaluation	
Motivation			
Automatic			
Reflective		Personalizability of home visit	
		Pay for LHWs	

*Abbreviations*: *COM-B* Capability, Opportunity, Motivation Determine Behavior Model, *LHWs* lay health workers, *TB* tuberculosis

^a^Capability represents the faculty to engage in a behavior and has a “physical” domain (e.g., strength, skills, stamina) and a “psychological” domain (e.g., knowledge, memory)

^b^Opportunity represents environmental factors that affect the capability to perform the behavior and has a “physical” domain (e.g., time, physical environment) and a “social” domain (e.g., interpersonal influences, social cues, cultural norms).

^c^Motivation represents the internal factors that allow one to employ capability and opportunity to perform a behavior, and has a “reflective” domain (e.g., beliefs and intentions) and an “automatic” domain (e.g., wants, needs, impulses)

Severe staffing shortages in TB units hindered health-center staff from doing community-based work, including contact investigation. At the clinics, health workers reported having several duties, especially in TB units, and competing responsibilities often left insufficient time to educate and counsel index patients effectively, much less to collect the information required to initiate contact investigation. Consequently, tasks like TB education and counselling, often viewed as being of a low priority were ignored. The contrast with HIV clinics was stark:

You want to pass on [information] but … we have limited staff…. For the HIV people…, they have their counsellors…, but it is unfortunate that [in] TB we don’t have specific counsellors…, so you find you are the one dispensing the TB medicine and you are also the one health-educating…, so [you] find there [are] many patients to talk to and therefore not enough time is spent on each patient…. (FGD, clinic staff)

A lack of designated, well-ventilated space for TB-related activities also hampered the ability to provide education and counselling in at least two ways. First, health workers expressed concerns about discussing sensitive personal health information around other patients. In addition, health-center staff sought to minimize the time spent with TB patients to reduce the risk of TB transmission:

It depends on how long you want to have a discussion with this patient in a closed room. I am not saying it is wrong to have discussions…, but you have to decrease the contact time with the patient, you have to take [only] the vital information. (FGD, clinic staff)

Finally, LHWs and health-center staff identified perceived stigma associated with TB and/or HIV as a key barrier to arranging household visits because it limited the ability of health workers to obtain details necessary for follow-up at home. Both groups reported that index patients feared loss of privacy and discrimination from household and community members if TB and/or HIV status were disclosed:

Some have stigma from people who think that all TB patients must have HIV. So, they do not want us to visit them because they fear you can disclose to other people that they have TB. (FGD, LHW)

All groups of stakeholders blamed stigma for avoidant behaviors among index patients, such as refusing to provide personal phone numbers or providing incorrect phone numbers and/or wrong directions to their homes. LHWs and health-center staff attributed TB-associated stigma to a lack of general knowledge in the community about TB, especially as compared with general knowledge about HIV. Furthermore, a lack of community involvement in TB health education seemed to worsen this gap. LHWs recommended wider dissemination of information about the benefits of home TB contact investigation:

…there should be some sensitization being done in the community, it can be in form of crusades, it can be TV, it can be radio…, let’s use the chairman [village leaders], let’s use the village health teams; they are more paramount than any health worker. (FGD, LHW)

### Arranging household visits through index TB patients: facilitators

The facilitators identified throughout the three key contact investigation activities are organized within the COM-B framework in Table 2B. Health-center staff described LHWs as critical for setting up household visits because of their ability, as lay members of the community, to bypass index patient mistrust of health workers, and the flexibility to visit households at times convenient to household contacts, such as nights and weekends. LHWs detailed how they engage treatment supporters by explaining the purpose of home visits and the dangers of TB transmission in the household. They emphasized the value of mobile phones for arranging and confirming home visits, especially for patients unable to provide clear directions or landmarks:

…you can never reach the [household] if [you are] directed without a phone…. You cannot go and just start asking; some [patients] are not known in their communities. (FGD, LHW)

### Visiting households to screen and refer contacts: barriers

A second principal activity of contact investigation is visiting households of index TB patients to screen contacts for TB symptoms. LHWs locate households, screen contacts for TB, and refer symptomatic and high-risk contacts to clinics for evaluation. Contacts and LHWs identified several barriers to completing home TB screening: (1) difficulty finding contacts present; (2) avoidant behavior among contacts related to TB- and HIV-associated stigma; (3) lack of TB knowledge among contacts, and (4) language barriers (especially in interacting with immigrants from neighboring countries, those speaking unfamiliar regional dialects, and the hearing- and/or speech-impaired).

… and whenever they are to come I have to prepare someone to help interpret…. (FGD, LHW)

Despite making and confirming most appointments by phone, LHWs often made multiple home visits to complete screening, or else scheduled weekend or early morning visits to increase the likelihood of finding contacts at home. However, this accommodation required LHWs to extend their working week. LHWs also reported avoidant behaviors among contacts, including turning LHWs away after arrival at a home for a scheduled visit and providing misleading answers to LHWs questions about TB symptoms that hinder successful identification of symptomatic contacts. LHWs attributed this elusiveness to denial, stigma, and/or a fear of contracting TB:

…they do not want to be in the same condition the [index] patient is in. (FGD, LHW)

We found that knowledge of TB among contacts varied widely. Contacts with the most accurate information about TB transmission were from households that LHWs had already visited. Many contacts, especially from households that had never had a LHW visit, had no knowledge about TB symptoms or TB transmission. However, they indicated a desire to learn:

We do not know TB symptoms and we shall be grateful if you told us about those symptoms today. (Interview, contact)

The lack of knowledge about the causes and consequences of TB meant that contacts were sometimes suspicious of LHWs visiting their homes, which they viewed as outside expected norms:

It is not the doctor to look for a patient but the patient to look for the doctor. (Interview, contact)

Last, to ensure success of contact investigation, LHWs reported it essential to remain inconspicuous during home visits:

…[Some] are afraid that we might go with company vehicles, making it possible for the rest of the village members get to know what they are suffering from. (FGD, LHW)

Household contacts concurred, suggesting that visits be conducted entirely indoors and/or that LHWs visit without visible identifiers such as uniforms and labelled vehicles, to reduce the risk of perceived or actual stigma.

### *Visiting households* to screen and refer contacts*: facilitators*

We identified two main categories of facilitators of household visits: (1) flexibility, privacy, and personalizability of home-based evaluation, as compared with clinic-based evaluation; and (2) financial support for LHWs. Health-center staff and LHWs, in particular, agreed that the ability of LHWs to coordinate and adapt services to the needs of contacts likely increased acceptance of contact investigation.

…if you have time and screen one-on-one, and ask in a friendly way and not do it as if you are investigating, but in a comfortable way, you get good response. (FGD, LHW)

The LHWs reported that collectively they identified their strengths and sometimes assigned tasks based on these. For example, some were more skilled in interacting with young clients while others excelled in attending to elderly ones.

*“…When you go, and find it a little difficult, you schedule for another appointment and go back along with another [LHW] who you are sure can handle such category of patient better.” (FGD, LHW)*

Contacts preferred the more leisurely pace of home visits, which provided the opportunity to ask questions and learn from LHWs:

…she taught me so many new things that I never knew and when she comes [to my] home, I get enough time to inquire about so many things. (Interview, contact)

Second, both health-center staff and LHWs emphasized that paying LHWs for the time and costs of home visits would signal to LHWs that this work is valuable, thereby incentivizing performance:

…let him have that extra [allowance], at least a top up for some of those extra activities…please make sure there is transport…lunch, let there be that airtime [for phone calls]. Let there be that extra work that I have done but let it be paid for in terms of money…. (FGD, clinic staff)

### Evaluating contacts for TB in clinics: barriers

The third activity for TB contact investigation is ensuring that contacts attend clinic to complete TB evaluation. Health workers and contacts described challenges to completing clinic evaluation, including: (1) contacts’ lack of money and time for travel; (2) inconvenient, unfriendly, and unresponsive clinic services; (3) fear of stigma among contacts; and (4) a lack of local contact investigation guidelines.

Health-center staff, LHWs, and contacts all noted the costs of getting to a clinic could be a major impediment to accessing free TB services, although some contacts described the additional effort they make to get to clinic:

Concerning health you can never fail to go; you try as much as possible [to get fare].… (Interview, contact)

On the other hand, both LHWs and contacts faulted inconvenient hours, long wait times, inattentive staff, and inadequate services for the reluctance of some contacts to attend clinics and for a broader distrust of the health system:

You know what really makes it hard for us is spending a lot of time at the clinic and leav[ing] without getting the treatment or medicine… (Interview, contact)

Contacts reported these long wait times related to health worker tardiness and absenteeism. LHWs also noted that many health workers are unfriendly:

The other problem that forces people not to come is the reception they receive at the clinic; there is a bad history about health workers which makes people fear to come. (FGD, LHW)

In addition, contacts expressed dissatisfaction with the quality of clinic services:

The other thing is when you go to the hospital and you tell them that you are suffering from cough, they won’t give you a lot of attention. They won’t take time to investigate what exactly you might be suffering from and they will just give you, say Panadol or Septrin, which you would have bought by yourself and not wasted time going to the hospital. (Interview, contact)

Furthermore, all participant groups mentioned fear of stigma associated with TB and HIV led contacts to delay clinic visits:

…people fear to know their health status, because you may go to the clinic and find someone who knows you, yet you don’t want them to know your status [TB and HIV]. That is also a big issue. (Interview, contact)

Finally, health workers expressed concern about the need to have explicit guidelines on how to conduct contact investigation:

We must have written guidelines on how to evaluate, so that anyone who receives a patient, is sure and knows what to do, don’t assume we all know. (FGD, clinic staff)

### Evaluating contacts for TB in clinics: facilitators

Stakeholders identified several observed and potential facilitators of clinic-based TB evaluation, including (1) physical and social support from family; (2) more streamlined and personalized contact evaluation services at clinics; and (3) education and social support from LHWs.

First, LHWs viewed family support as an important element of effective clinic-based evaluation:

When you find the contacts at home and [they] are very weak, family members help them a lot especially when they [contacts] are coming to the hospital. (FGD, LHW)

…within that household [contacts] would have treatment support in terms of encouragement… to take drugs and … diet related… care like in terms of understanding them…so in terms of that support they give. (FGD, LHW)

LHWs also viewed reduced clinic waiting times and streamlined evaluation as important incentives that clinics should prioritize:

…we know these people are from the community. They have just been persuaded to come…. So, you [should] give them at least first priority.... (FGD, LHW)

Finally, contacts found counselling from LHWs motivating:

If they have explained very well like the community worker told us if you have the sickness and you start the medication early, you get better faster… I wouldn’t wait… I would want to get healed and wouldn’t want to be sick for a long time. I would run to the health unit for treatment because she explained [that] very well to me…. (Interview, contact)

### Classifying barriers in behavioral terms and identifying potential intervention functions

Using the COM-B model, we categorized the above stakeholder-reported barriers to and facilitators of household contact investigation activities in terms of their behavioral determinants (Table 2A and B) and formulated a “behavioral diagnosis” for the identified barriers. For example, the lack of a designated and well ventilated space for health workers to educate and counsel TB patients, represents a physical opportunity barrier.

Stakeholders identified several modifiable barriers and facilitators that we were able to link to appropriate intervention functions using the BCW framework (Table 3). For example, multiple informants highlighted the importance of enhancing knowledge of TB among contacts through *education* and the importance of providing emotionally compelling information about health consequences through *persuasion*. Another intervention function identified was *enablement*, physical and emotional support provided by LHWs or family members to help index contacts complete clinic visits. We also identified the value of the intervention function of *incentivization* in the recommendation of health-center staff that LHWs be paid for their work, and in the suggestion from LHWs that streamlining clinic visits could better motivate contacts to attend clinic visits. In addition, multiple participants voiced a need for *environmental restructuring* interventions, such as creating private clinic spaces for counselling and shifting contact evaluation from the clinic to the community. Finally, although not mentioned by stakeholders, we identified *modeling* in the form of testimonials from former TB patients as another potentially effective intervention function targeting social detachment and de-motivation linked to stigma. A summary of selected intervention functions that would address the behavioral barriers and enhance the facilitators is provided in Tables 4 and 5.

**Table 3:** Investigator-identified Intervention functions targeting identified barriers and facilitators as defined in the Behavioral Change Wheel framework

Intervention Functions	Arranging home visits	Visiting households to screen contacts	Evaluating contacts in clinics
Education	LHWs use their lay understanding of community knowledge gaps and concerns to counsel patients		
Training			
Persuasion	LHWs establish trust with index patients		
	LHWs convince index patients to accept CI		
Environmental restructuring		Home visit allows scheduling flexibility	Clinic-staff streamline clinic visits for contacts
Enablement	LHWs give social support to index patients	Home visit provides privacy	Families support contacts physically
	Clinic-staff shift task of visiting home to LHWs	Contacts trust LHWs	Families support contacts emotionally
	CHWs call index patients on mobile phones		
Modeling	Former TB patients recount their experiences in referring their contacts		
Incentivization	Clinic funds LHW transport fare	Home visit eliminates some clinic visits	Clinic-staff streamline clinic visits for contacts
		Clinic or program pays LHWs for their work	
Restriction			
Coercion			

*Abbreviations*: *LHWs* lay health workers, *CI* contact investigation, *TB* tuberculosis

**Table 4:**
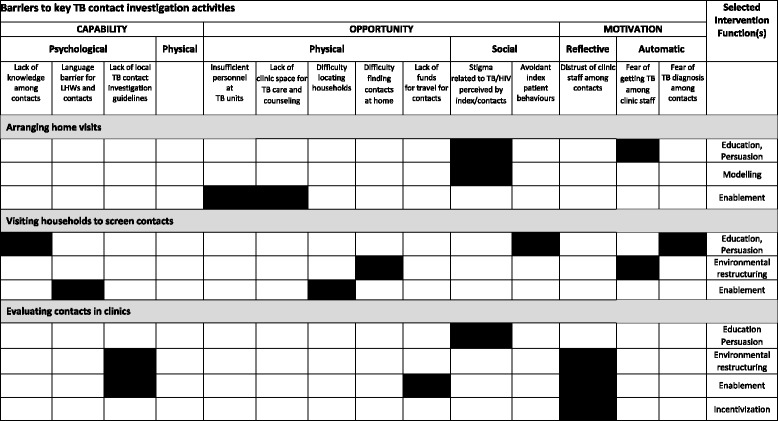
Summary of modifiable barriers and selected linked intervention functions

Legend: Shaded cells identify intervention functions that best target the identified barriers, after applying the Behavior Change Wheel framework.

**Table 5:**
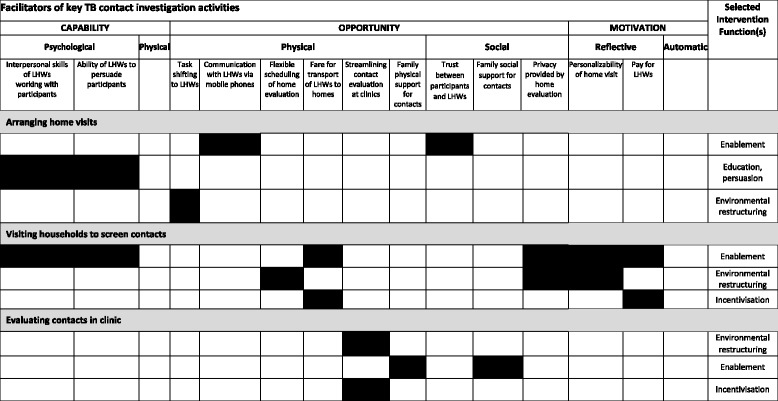
Summary of selected facilitators and linked intervention functions

Legend: Shaded cells identify intervention functions that best target the identified facilitators, after applying the Behavior Change Wheel framework

## Discussion

The 2015 global End TB Strategy [4] calls for bold, new, patient-centered, active case-finding strategies. Changing the behavior of providers and patients will be critical for any of these new approaches to succeed. In pursuit of these goals, we systematically engaged front-line stakeholder communities in open-ended discussions about barriers to and facilitators of household contact investigation in Kampala, Uganda. We applied qualitative methods, behavioral theory, and a comprehensive behavior change framework to understand and respond to patient and health system factors to better enable this prototypical approach to community-based active TB case-finding.

The behavioral determinants we identified were wide-ranging and covered all three domains of the COM-B model. Informants clearly described a need for information about TB to enhance not only clinical knowledge but also knowledge about how household members relate to the possibility of undiagnosed illness and seek care in response. These needs of community members appeared to be mediated by social influences such as fear of stigma and by environmental factors, including a lack of time and space to obtain information and build rapport in clinics, and long travel and wait times to reach clinics resulting in high costs to seeking care. Participants further described how, in turn, fears about health and social outcomes of contact evaluation, as well as mistrust of and mistreatment by health center staff, frequently deter index patients and contacts from willingly participating in contact investigation.

The main facilitator of contact investigation that we identified was the involvement of LHWs themselves in providing personalized and enabling services to index patients and contacts, from the clinic to the home and back. All three groups of stakeholders repeatedly cited the value that LHWs add to the contact investigation process and highlighted the need to pay them and provide adequate financial resources to facilitate home visits. This adds to a modest literature describing a varied and context-specific role of LHWs that is largely effective in improving delivery of TB care and enhancing health outcomes, and emphasizes the critically important policy message that LHWs should be paid [36]. Our study also provides compelling examples of *how* LHWs can modify important behavioral determinants of key contact investigation activities, and application of the BCW implementation framework enabled us to link to a variety of possible intervention functions to directly address these. These included educating and persuading participants; restructuring the physical environment; and modeling, incentivizing, and otherwise enabling health-positive behaviors—usually in close cooperation with health-center staff, LHWs, participant family members, and with use of mobile phone communications and/or the media. Of note, none of the stakeholders identified a lack of physical skills or a need for training to enhance those skills. This emphasizes their view that while contact investigation is a complex intervention, it simply requires that they adapt existing skills to work in a new context rather than that they acquire entirely new ones.

Many of these findings have been previously reported in other settings. Fox and colleagues carried out a nested case-control study of household contacts comparing knowledge, attitudes, and practices related to TB and contact investigation among clinic attenders and non-attenders in Vietnam [37]. Although the structure, location, and timing of contact investigation activities differed from our study, contacts in Vietnam also found invitations to test for TB acceptable; described discrimination and stigma as major barriers to uptake; and described time constraints as a major barrier to completing clinic evaluation. Another study from Thailand surveyed and interviewed index patients to identify predictors of their contacts attending clinic evaluation [38]. These authors identified travel distance; lack of permission to bring children of other household contacts for evaluation; and perceived susceptibility of contacts to TB, perceived barriers to clinic follow-up, and intention to bring contacts to clinic as predictors of contacts coming to clinic. Finally, a series of case studies compiled from a variety of settings in Africa and Asia in the TB CARE Contact Investigation Implementation and Adaptation Guide [11] described a similar set of behavioral determinants among contacts. These included a lack of TB knowledge, alternative cultural models for understanding TB, and forgetting appointments; long distances to clinic and lack of time to travel; discrimination; fear of specific aspects of evaluation; and preferences for evaluation in specific settings and reluctance to visit under-resourced clinics. However, by seeking perspectives from health-center staff and LHWs in addition to contacts, we have identified previously under-emphasized barriers to contact investigation and potential interventions to improve its efficiency. Most notable among these are limitations in the psychological capacity of participants to engage in contact investigation and in their physical opportunity to complete contact investigation procedures. Specifically, front-line stakeholders all highlight the need for more holistic TB education, counselling, and community sensitization interventions that address not only the biology of TB and the logistics of treatment, but also the human experience of transmitting or acquiring a deadly and stigmatized infection from a household member. In addition, stakeholders emphasized the need to restructure TB evaluation services to overcome the barriers of time, space, distance, and mistrust of health center staff. Finally, all stakeholders were receptive to lay health workers playing an integrative role in addressing these needs through more patient-centered, home-based TB evaluation services.

Our study has several strengths. First, we have applied several core principles of implementation science: identifying an important evidence-practice gap, engaging communities, carrying out formative research, and analyzing our data using a validated theory of change [17]. Our rigorous behavioral analyses applied to our data and to the published literature show the value and flexibility of a systematic, comprehensive, and coherent approach to identifying determinants of behaviors. Finally, our use of a linked implementation framework demonstrates how to begin tailoring interventions to identified barriers.

Our study had some limitations. As baseline, formative data, there is no link to outcomes. Data collection was cross-sectional, limiting our ability to explicitly verify our findings and further explore emergent themes including health worker knowledge and attitudes about TB; which could explain their fears of contacting TB. Finally, we did not include index patients in these interviews.

## Conclusions

Using a comprehensive theoretical approach, we systematically identified determinants of behaviors relevant to key TB contact investigation activities in our setting that may be useful in tailoring interventions to improve implementation of contact investigation in Kampala and similar urban settings. In addition, we have laid a strong foundation for the development and implementation of interventions for active TB case finding and for identifying appropriate behavior change techniques to deliver them.

## Additional file

Additional file 1: Supplementary materials, including Supplemental Methods and Appendices containing Focus Group Discussion and Interview Guides. (DOCX 36 kb)
