# Supplementary material for: Identifying barriers to and facilitators of tuberculosis contact investigation in Kampala, Uganda: a behavioral approach
Source: Implement Sci. 2017 Mar 9;12:33. doi: 10.1186/s13012-017-0561-4 (PMC5343292; doi:10.1186/s13012-017-0561-4)
Supplement: Supplementary file 1 — Supplementary materials, including Supplemental Methods and Appendices containing Focus Group Discussion and Interview Guides. (DOCX 36 kb) [file 13012_2017_561_MOESM1_ESM.docx]

**Supplemental Methods**

We developed a hierarchical coding system to guide analysis of the interview and focus group data that we collected. We started by specifying parent codes for each of the three target activities: screening index patients; visiting households for TB screening; household contacts returning to clinic for TB evaluation. Second, we developed child codes inductively based on emergent themes identified by the primary coders. An experienced social scientist reviewed the coding scheme and approved the coding scheme. Two primary coders (I.A., J.G.) reviewed all transcripts; disagreements were resolved by discussion and referral to the study investigators with expertise in coding qualitative data.

**Appendices**

1. Health Care Worker Focus Group Discussion Topic Guide
2. Community Health Worker Focus Group Discussion Topic Guide
3. Household Contact Interview/Focus Group Discussion Topic Guide

**Systematic Screening for TB in Households of TB Patients (TB Contact Investigation) Implementation Study**

I’d like to start by asking each one of you to state your name slowly to help the person who will be transcribing the interview recognize your voice.

The topic for today’s discussion is « tuberculosis (TB) contact investigation», which includes systematic screening (“investigation”) for TB among household members (“contacts”) of new patients with active TB. The World Health Organization recently issued recommendations that TB contact investigation be performed more widely. We are working with the Uganda NTLP and the KCCA to understand how to implement the guidelines in Uganda. The information from this focus group will help us to learn from you how best to implement the guidelines and to develop appropriate training materials for different groups of clinicians and HCWs.

1. I would like to start by asking you what you think the risk of TB is among people living in the same household as a patient diagnosed with active TB. [KNOWLEDGE]
2. Have you ever spoken with your TB patients about the possibility that others in their household are also at risk of having TB? [OPPORTUNITY].
3. The WHO recommendations suggest that a list of household contacts should be obtained from all TB patients. How possible/feasible would it be to do this in your clinic? [PROCESS 1].
4. The WHO recommendations state that someone from the clinic should visit the household to screen all listed household members for TB symptoms. How feasible/possible would it be to do this in your clinic? [PROCESS 2]
5. The WHO recommendations state that household members, who have TB symptoms, are young children, or who have HIV infection should present to the clinic and be evaluated for TB. [PROCESS 3]
6. How possible/ feasible would it be to evaluate household members who have TB symptoms in the clinic?
7. How possible/ feasible would it be to evaluate young children for TB in the clinic?
8. How possible /feasible would it be to evaluate household members who have HIV infection in the clinic?
9. Now that you have heard about contact investigation, how would you feel about your clinic participating in such a program? [MOTIVATION]

That is all the questions we have for you today. Is there anything else that YOU think is important about this topic that we haven’t asked about?

Overall, what were your thoughts about the interview?

**Prompts and Follow-up Questions**

This side provide specific prompts and/or follow-up questions to help focus and/or redirect respondents as needed to make sure that they are addressing the issues we are interested in exploring/

1. [KNOWLEDGE] No specific Prompts/Follow-up.
2. [OPPORTUNITY] “Speaking with patients about TB”

Prompts/Follow-up:

1. If yes, what do you tell them?
2. If no, what factors prevent you from having such discussions?
3. Do you think there are any harms to discussing the risk of TB among other people living in their household? What about any benefits?
4. [PROCESS 1] “Enumerate contacts”

Prompts/Follow-up:

1. Who would be the best person to obtain the list of household contacts?
2. When would it be done?
3. How and where would the list of contacts be recorded?
4. [PROCESS 2] “Visit index household”

Prompts/Follow-up:

1. Are there clinic workers or volunteers who already conduct household visits for other clinic programs? How do they get there? Are they paid for these activities?
2. If your clinic was to take up contact investigation, who should be responsible for visiting households to screen household members for TB symptoms?
3. How would this person or persons obtain the list of households that need to be visited?
4. [PROCESS 3] “Evaluate contacts in clinic”

Prompts/Follow-up:

- 1. How would they be identified as a household member of a TB patient?
  2. Could their evaluation be expedited? If so, how?
  3. How would young children be evaluated for TB?

6. [MOTIVATION] “Overall feelings about contact investigation”

Prompts/Follow-up:

1. Are there other activities that you think the clinic should be doing that are of higher priority?
2. What would be the next steps to get such a program started in your clinic? Is there anything that would help to start such a program?

**Systematic Screening for TB in Households of TB Patients (TB Contact Investigation)**

**Implementation Program**

We would like to talk with you today about your experiences with tuberculosis (TB) contact investigation, which is when CHWs interview household members of a TB patient to see if they have symptoms or risk factors that require testing or evaluation by a clinician for TB. We are working together with the Uganda National TB and Leprosy Programme (NTLP) and Ministry of Health (MoH) personnel from the Kampala Capital City Authority (KCCA) clinics to evaluate how well this activity is going and to see if we can identify ways to improve it. The information that you share in this focus group will help us learn how best to do this.

1. We understand that household contact investigation is part of Track TB’s current activities. Would you tell us how you conduct a household TB contact investigation? Start with identifying the index patient at the clinic and finish with any follow-up you are required to do after screening all of the household contacts? Would you also specifically describe the last contact investigation you performed? [OVERALL PROCESS]
2. How agreeable are index patients to allowing you to visit their homes? [PROCESS 1]
3. How easy or difficult is it to find the houses of index patients? How easy or difficult is it to find the household contacts at home and willing to be screened for TB? [OPPORTUNITY]
4. How easy or difficult is it to interview household contacts about TB symptoms? [PROCESS 2]
5. How easy or difficult is it to get household contacts to come to clinic to be evaluated? How successful are you in tracking whether they actually have come to clinic or not? How, if applicable, do use phone or SMS? [PROCESS 3]
6. What, if any, additional knowledge or skills would help you improve your ability to do household contact investigation? Do you have any experience collecting sputum? Doing HIV counseling and testing? Using tablets to collect data? [KNOWLEDGE]
7. Do you feel that contact investigation is something that we should continue to do? Would you be interested in helping evaluate new ways to improve the process? Why or why not? [MOTIVATION]
8. Would you be willing to collect sputum in the home? Do HIV counseling and testing? Record results in a tablet? Why or why not? [MOTIVATION]

That is all the questions we have for you today. Is there anything else that YOU think is important about this topic that we haven’t asked about?

Overall, what were your thoughts about the interview?

**Prompts and Follow-up Questions**

This side provide specific prompts and/or follow-up questions to help focus and/or redirect respondents as needed to make sure that they are addressing the issues we are interested in exploring/

1. [OVERALL PROCESS] “Contact Investigation Experience”

Prompts/Follow-up:

What are the individual steps that you and your team follow?

1. [KNOWLEDGE] “About Contact Investigation”

Prompts/Follow-up:

What additional training do you feel that you would need (*e.g.* training to screen HIV-positives or children)?

1. [PROCESS 1] “Index Patients”

Prompts/Follow-up:

What would have made this easier? / What would have made it difficult?

1. [OPPORTUNITY] “Finding the Household”

Prompts/Follow-up:

What challenges have you faced? How have you solved them?

1. [PROCESS 2] “Interviewing Contacts about Symptoms”

Prompts/Follow-up:

What approaches are not working well? What approaches are working successfully?

1. [PROCESS 3] “Contact Follow-Up”

Prompts/Follow-up:

What approaches are not working well? What approaches are working successfully?

1. [MOTIVATION] “Overall Feelings About Contact Investigation”

Prompts/Follow-up:

How does contact investigation compare with other activities you have done at the clinic?

1. [MOTIVATION] “Willingness to adopt new procedures”

Prompts/Follow-up:

How does contact investigation compare with other activities you have done at the clinic?

**Systematic Screening for TB in Households of TB Patients (TB Contact Investigation)**

**Implementation Program**

The topic for today’s discussion is “Systematic Screening for TB in Households of TB Patients.” This is when CHWs visit the homes of community members who have a TB patient in their household to see interview them to determine whether others in the household have TB symptoms and might also have TB disease. We are working with the Uganda National TB and Leprosy Programme (NTLP) and with Ministry of Health (MoH) personnel from the Kampala Capital City Authority (KCCA) clinics to evaluate how well this activity is going and to learn how we can improve it using new approaches. The information that you share in this focus group will help us do this.

1. Have any health workers from the Track TB Program recently visited your home to provide information and support for TB care for your household? What happened when they came to your house? [PROCESS 1]
2. What you think the risk of TB is among people living in the same household as a patient diagnosed with active TB? What are the chances that someone who has symptoms of TB also has HIV? [KNOWLEDGE]
3. How easy or difficult was it (or would it have been) for you to go to the clinic to be tested and evaluated for TB, if you had been (or ever were in the future) asked to do so by the community health worker? [OPPORTUNITY, MOTIVATION]
4. What did you think of the care and services that you received (or do you think of the services there in general)? [PROCESS 2]
5. If you needed it, how would you feel about our collecting a sputum specimen from you at home rather than having you come to clinic to give that specimen? [PROCESS 1a] How would you feel about our offering you voluntary HIV counseling and testing in your home? [PROCESS 1b]
6. How would you feel about our recording information from you using an electronic tablet? How would you feel about our scanning your fingerprint to help us track your visits to the clinic? [PROCESS 0]

Now we are going to take a break from the discussion. We are going to ask you a few questions individually about your use of mobile phones. We will then finish the discussion by talking about your answers together.

1. How would you feel if we sent you an SMS with the results of your sputum examination and advice about the need to come to clinic for additional evaluation or treatment, instead of having you go back to clinic to get this information or having a health worker come to deliver it? Which words do you prefer for the sample SMS included in the survey? [PROCESS 1c]

That is all the questions we have for you today. Is there anything else that YOU think is important about this topic that we haven’t asked about?

Overall, what were your thoughts about the interview?

**Prompts and Follow-up Questions**

This side provide specific prompts and/or follow-up questions to help focus and/or redirect respondents *as needed* to make sure that they are addressing the issues we are interested in exploring.

1. [PROCESS 1] “Have CHW come to ask about TB at home”

Prompts/Follow-up:

How did you feel /would you feel about having a CHW come into your home to interview you about TB symptoms?

1. [KNOWLEDGE] “Risk of TB in the home”, “Risk of HIV in someone who has symptoms of TB”

Prompts/Follow-up:

Please draw on information you received from the community health worker, and on your general understanding of TB.

1. [OPPORTUNITY, MOTIVATION] “Traveling to the clinic”

Prompts/Follow-up:

1. What would have made this easier?
2. Difficulties finding time or funds to take transport?
3. [PROCESS 2] “Visiting the clinic”

Prompts/Follow-up:

Is there anything the health workers could have done to make this easier?

1. [PROCESS 1a, PROCESS 1b] “Providing sputum at home”, “HIV Testing at Home”

Prompts/Follow-up:

- 1. What concerns, if any, would you have about the safety of this?
  2. What concerns, if any, would you have about standing outside your home to do this?

1. [PROCESS 0] “Providing personal health information and fingerprint scan to tablet”

Prompts/Follow-up:

Would you have any privacy concerns?

1. [PROCESS 1c] “Receiving text messages”

Prompts/Follow-up:

1. Show sample SMS content and ask for feedback on wording. Which messages do you prefer?
2. Is there anything we could do to address these concerns (*e.g.* requiring a password to access an SMS?)
3. If you are unable to read, how acceptable would it be for someone else to help you read SMS notifications on your phone? Would you have any privacy concerns, for example if the SMS include personal health details?
